# Rare genetic variation at transcription factor binding sites modulates local DNA methylation profiles

**DOI:** 10.1371/journal.pgen.1009189

**Published:** 2020-11-20

**Authors:** Alejandro Martin-Trujillo, Nihir Patel, Felix Richter, Bharati Jadhav, Paras Garg, Sarah U. Morton, David M. McKean, Steven R. DePalma, Elizabeth Goldmuntz, Dorota Gruber, Richard Kim, Jane W. Newburger, George A. Porter, Alessandro Giardini, Daniel Bernstein, Martin Tristani-Firouzi, Jonathan G. Seidman, Christine E. Seidman, Wendy K. Chung, Bruce D. Gelb, Andrew J. Sharp

**Affiliations:** 1 The Mindich Child Health and Development Institute and Department of Genetics & Genomic Sciences, Icahn School of Medicine at Mount Sinai, New York, New York, United States of America; 2 Department of Newborn Medicine, Boston Children’s Hospital, Boston, Massachusetts, United States of America; 3 Division of Cardiovascular Medicine, Brigham and Women’s Hospital, Boston, Massachusetts, United States of America; 4 Department of Genetics, Harvard Medical School, Boston, Massachusetts, United States of America; 5 Howard Hughes Medical Institute, Harvard University, Boston, Massachusetts, United States of America; 6 Division of Cardiology, Children’s Hospital of Philadelphia, Philadelphia, PA, United States of America; 7 Department of Pediatrics, University of Pennsylvania Perlman School of Medicine, Philadelphia, PA, United States of America; 8 Department of Pediatrics, Cohen Children’s Medical Center, Northwell Health, New Hyde Park, NY, Unites States of America; 9 Department of Pediatrics, Keck School of Medicine, University of Southern California, Los Angeles, California, United States of America; 10 Department of Cardiology, Boston Children's Hospital, Boston, Massachusetts, United States of America; 11 Department of Pediatrics, Harvard Medical School, Boston, Massachusetts, United States of America; 12 Department of Pediatrics, University of Rochester Medical Center, Rochester, NY, United States of America; 13 Cardiothoracic Unit, Great Ormond Street Hospital, London, England; 14 Department of Pediatrics, Stanford University School of Medicine, Stanford, CA, United States of America; 15 Department of Pediatrics, University of Utah School of Medicine, Salt Lake City, UT, United States of America; 16 Departments of Pediatrics and Medicine, Columbia University, New York, NY, United States of America; 17 Department of Pediatrics, Icahn School of Medicine at Mount Sinai, New York, United States of America; 18 Graduate School of Biomedical Sciences, Icahn School of Medicine at Mount Sinai, New York, New York, United States of America; University of Bristol, UNITED KINGDOM

## Abstract

Although DNA methylation is the best characterized epigenetic mark, the mechanism by which it is targeted to specific regions in the genome remains unclear. Recent studies have revealed that local DNA methylation profiles might be dictated by *cis-*regulatory DNA sequences that mainly operate via DNA-binding factors. Consistent with this finding, we have recently shown that disruption of CTCF-binding sites by rare single nucleotide variants (SNVs) can underlie *cis*-linked DNA methylation changes in patients with congenital anomalies. These data raise the hypothesis that rare genetic variation at transcription factor binding sites (TFBSs) might contribute to local DNA methylation patterning.

In this work, by combining blood genome-wide DNA methylation profiles, whole genome sequencing-derived SNVs from 247 unrelated individuals along with 133 predicted TFBS motifs derived from ENCODE ChIP-Seq data, we observed an association between the disruption of binding sites for multiple TFs by rare SNVs and extreme DNA methylation values at both local and, to a lesser extent, distant CpGs. While the majority of these changes affected only single CpGs, 24% were associated with multiple outlier CpGs within ±1kb of the disrupted TFBS. Interestingly, disruption of functionally constrained sites within TF motifs lead to larger DNA methylation changes at nearby CpG sites. Altogether, these findings suggest that rare SNVs at TFBS negatively influence TF-DNA binding, which can lead to an altered local DNA methylation profile. Furthermore, subsequent integration of DNA methylation and RNA-Seq profiles from cardiac tissues enabled us to observe an association between rare SNV-directed DNA methylation and outlier expression of nearby genes.

In conclusion, our findings not only provide insights into the effect of rare genetic variation at TFBS on shaping local DNA methylation and its consequences on genome regulation, but also provide a rationale to incorporate DNA methylation data to interpret the functional role of rare variants.

## Author summary

One of the major challenges for human genetics in the post-genomic era is to interpret the functional relevance of genetic variation. Quantitative trait locus (QTL) analyses have associated an important fraction of genetic variants with a wide range of molecular phenotypes including gene expression (eQTL) and DNA methylation (meQTL), providing insights into the mechanisms by which genetic variation can contribute to health and disease. Although QTL mapping represents an excellent approach to identify biologically relevant functional variants, these studies have been mainly focused on common variants and do not include low-frequency and rare variants. Here, we observed that rare regulatory variants, *i*.*e*, single nucleotide variants (SNVs) that disrupt transcription factor binding sites (TFBSs), are associated with changes in DNA methylation at both local and, to a lesser extent, broader locations, most likely, by altering the DNA-binding affinity of transcription factors (TFs). Interestingly, we have also shown that this change in DNA methylation can alter expression levels of nearby genes. Overall, these data suggest a role of rare regulatory SNVs in shaping DNA methylation, and suggest that the incorporation of DNA methylation data may help to interpret the functional consequences of human genetic variation.

## Introduction

Over the last decade, genomic DNA sequence variation has been associated with quantitative changes in multiple molecular phenotypes, including variation in gene expression and epigenetic marks such as chromatin accessibility and DNA methylation [1–5]. These variants are commonly referred to as quantitative trait loci (QTLs), the mapping of which has allowed us to unravel the primary mechanisms by which cis-regulatory variants can influence phenotypic variation. Single nucleotide variants (SNVs) present within cis-regulatory regions such as transcription factor binding sites (TFBSs) are enriched for different types of QTLs, such as those influencing chromatin structure/accessibility [1,2] and DNA methylation (meQTL) [3], suggesting these as functional regulatory variants. However, to date, most studies have focused on common variants, and the functional consequences of rare variants (minor allele frequency (MAF) ≤1%) have not been systematically interrogated.

Large population-scale projects using deep sequencing have provided extensive catalogues of human genomic variation, showing that rare genetic variants are abundant in the human genome [6–8]. It has been hypothesized that rare variants likely also contribute to the genetic architecture of complex diseases, which cannot be entirely explained by common genetic variation. Recent studies have demonstrated that some rare SNVs induce dramatic changes to the expression levels of nearby genes [9–11], which likely contribute to phenotypic variation. However, there is limited insight about how these variants can regulate these transcriptional changes. An important regulatory mechanism for controlling transcriptional activity is DNA methylation [12].

DNA methylation is an epigenetic modification crucial for mammalian development, playing a critical role in many cellular processes such as X-chromosome inactivation [13], genomic imprinting or maintenance of genome stability [12,14]. Although DNA methylation has been extensively characterized in mammals, the mechanisms by which it is targeted to specific genomic regions remains unclear. Recent data have shown that cis-acting methylation-determining regions (MDRs), short motifs that appear to encode epigenetic patterns in the local region, are sufficient for the *de novo* establishment of local DNA methylation patterns during embryonic development [15]. Interestingly, deleterious mutations at DNA binding motifs within these MDRs result in methylation changes at the adjacent CpGs. Furthermore, in line with this, we have recently shown that single base mutations at the canonical binding site for CCCTC-Binding Factor (CTCF) are enriched around cis-linked DNA methylation changes in both patients with congenital anomalies (CA) and in the normal population [16]. These findings suggest the potential for regulatory genetic variation to have profound effects on individual epigenetic profiles. However, it is still unknown whether this effect is limited to a few TFs or is a more general mechanism underlying the regulation of DNA methylation. Supporting the latter, experiments including targeted gene disruption of TFs in mouse stem cells have implicated DNA-binding factors in the regulation of local DNA methylation profiles [17]. Altogether, these data support an emerging role of DNA-binding TFs in regulating local DNA methylation profiles and suggest that this is likely influenced by genetic variation at their transcription factor binding sites (TFBSs).

Based on the potential role of cis-regulatory elements in modulating DNA methylation profiles locally [15,16], we hypothesized that the disruption of TFBSs by rare SNVs can modify local DNA methylation profiles and lead to altered transcription of nearby genes. To test this hypothesis, we combined blood DNA methylation profiles and SNVs derived from whole genome sequencing (WGS) of 247 unrelated individuals together with TFBS motifs for 133 different TFs predicted from chromatin immunoprecipitation sequencing (ChIP-Seq) data [18,19]. Subsequent integration of transcriptome and DNA methylation profiles from cardiac tissue enabled the study of the functional consequences of the TF-mediated DNA methylation at the transcriptional level.

## Results

### Rare variants at canonical TF motifs affect local DNA methylation profiles

**Fig 1:**
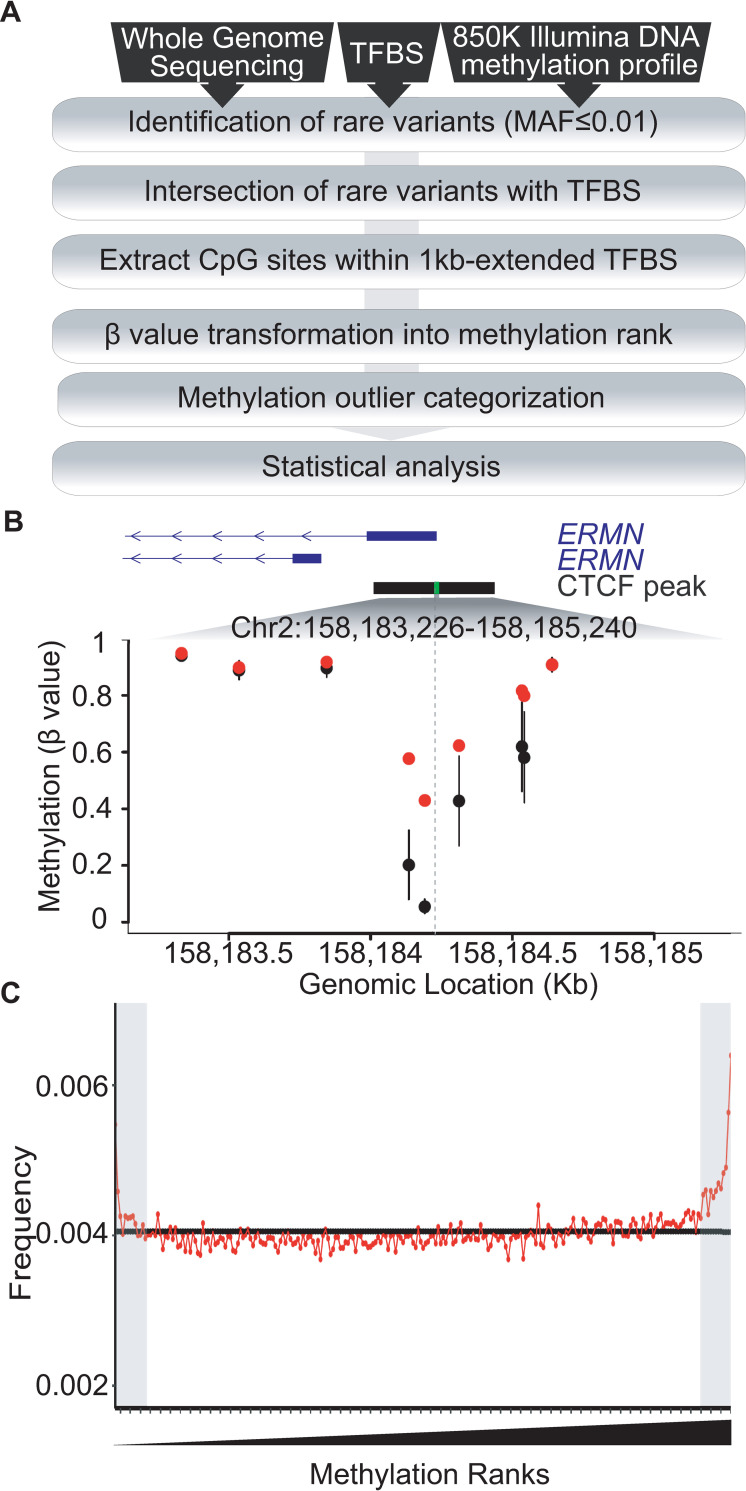
Effect of regulatory rare variants on shaping local DNA methylation profiles. (A). Analysis pipeline of the study. Briefly, after identifying rare variants that overlap TFBSs, we extracted DNA methylation values corresponding to CpGs that fell within the targeted TFBSs and their extended flanks (±1 kb) present on the EPIC array. For each CpG site, DNA methylation values were transformed into ranks across all individuals (n = 247) in ascending order, ranging from 1 to 247. Thus, DNA methylation values rise through the ranks, with the minimum and the maximum DNA methylation values in our cohort represented by the lowest and highest ranks, respectively. CpGs showing both DNA methylation ranks present into the 5% tails of the distribution and DNA methylation difference of ≥0.05 (β-value) as compared to the mean of controls, *i*.*e*, non-carriers for the tested rare variant, were considered as differentially methylated (also referred as DNA methylation outlier). (B) Plot showing DNA methylation profile at chr2:158,183,226–158,185,240 (CTCF binding site ±1 kb). Red dots indicate DNA methylation values for individual carrying rare variant (chr2:158,184,228), while black dots and bars represents the mean and mean ±2 standard deviation of controls, respectively. Disrupted CTCF motif (chr2:158,184,226–158,184,240) is highlighted in light green within the CTCF ChIP-peak (black box). Position of rare variant is depicted by vertical gray dashed line. (C) Plot showing distribution of DNA methylation ranks across population for all tested TFBSs (n = 133). Red dots represent the frequency of the rank for individuals carrying the rare variant, while the black horizontal line is the frequency in controls. Peaks at gray shaded areas of the graph represent an excess of individuals with extreme DNA methylation values for a given CpG site.

The flow diagram presented in Fig 1A represents our approach to integrate, filter and analyze data to determine the influence of rare regulatory genetic variation on local DNA methylation profiles. Briefly, after identification of rare SNVs that lie within TFBSs, we extracted β-values corresponding to the Illumina Infinium MethylationEPIC BeadChip (EPIC) microarray probes located within the proximity of the SNV-disrupted TFBSs and ranked them from lowest (1) to highest (247) (see methods for further details). Following this approach, we expected to identify rare regulatory SNVs that result in extreme high or low DNA methylation values in individuals carrying the tested SNV as compared to controls, *i*.*e*, individuals who do not carry the tested variant (Fig 1B).

The intersection between our genotype and DNA methylation datasets yielded a total of 127,335 SNV:TFBS pairs including 91,356 rare SNVs (MAF≤1%) within 120,096 different TFBS motifs that were informative for DNA methylation, *i*.*e*., EPIC probes mapping within 1 kilobase (kb)- flanks of SNV-targeted TFBS (S1 Table). To detect the effects of rare genetic variation on local DNA methylation profiles, we focused on the 5% tails of the rank distribution that showed an absolute minimum β-value difference of 0.05 between SNV carriers and controls. We observed that carriers of rare SNVs that disrupt TFBSs (SNV-TFBSs) show an increased burden of extreme methylation values as compared to individuals without rare variants (Fig 1C), suggesting a potential effect of rare regulatory SNVs in modulating local DNA methylation profiles.

To determine the extent of the effect of rare genetic variation within TFBS on DNA methylation, we explored whether a single or multiple CpG sites show extreme methylation values in the proximity of the mutated TFBS. Here, we considered those TFBS with 3 or more CpG sites located within 1kb and, at least one of them showing extreme methylation (n = 16,708). Of these, we observed that 12,682 (76%) had only a single outlier CpG, while 4,026 (24%) had between 2 and 13 CpG sites within 1kb with outlier DNA methylation values. To formally assess whether loci with outlier DNA methylation could be identified using an analysis that searches for regions containing multiple outlier CpGs, termed differentially methylated regions (DMRs), we performed a prospective screen for DMRs in each sample using a sliding window algorithm, similar to that described in Barbosa *et al*. [16]. Using this approach, we identified a total of 9,689 different DMRs in our cohort (median of 202 DMRs per sample). Because our algorithm was only able to detect DMRs at loci containing ≥3 probes within a 1kb interval, we intersected our DMR list with the set of 16,708 SNV-TFBS described above, and observed 744 loci (4.5%) of SNV-TFBS that showed a DMR within 1kb of the mutated TFBS (S2 Table).

**Fig 2:**
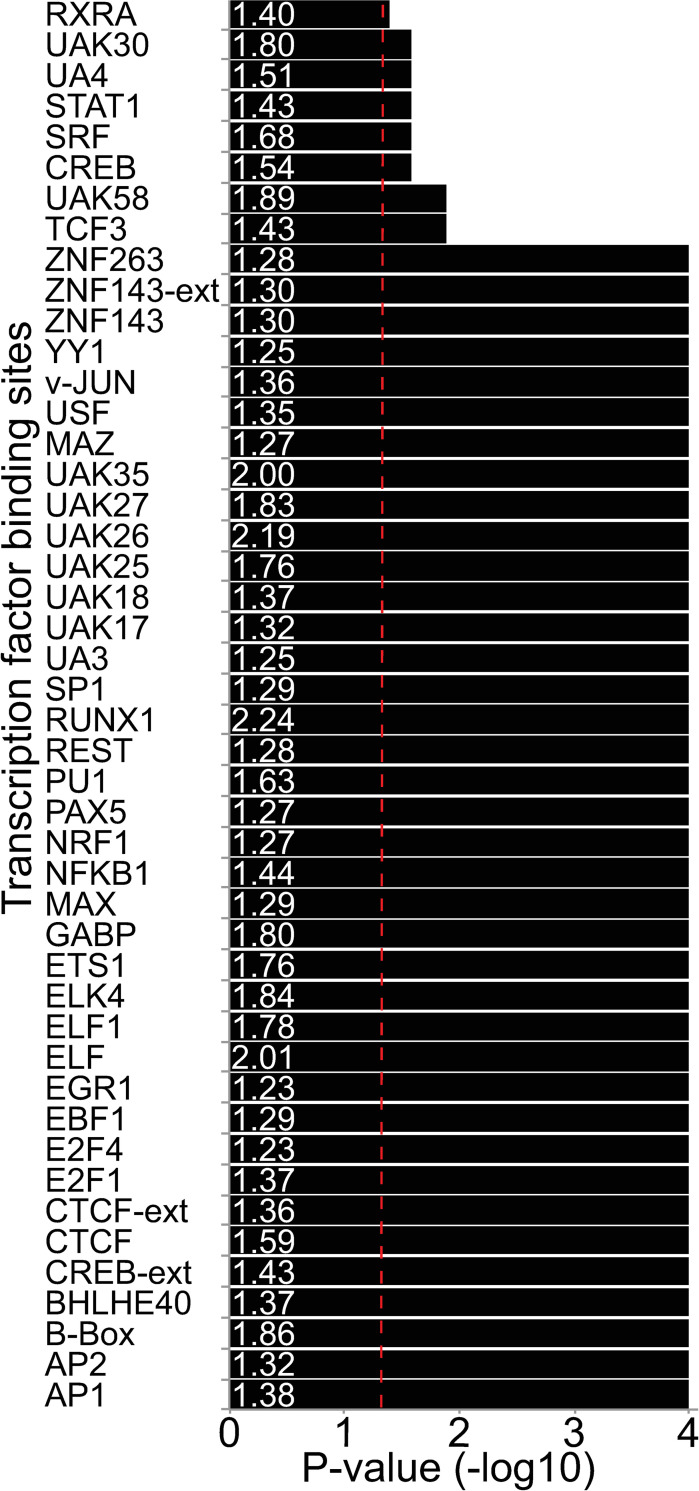
TFBS associated with local DNA methylation profile. Bars indicate P-values obtained for 46 TFBSs that were significantly enriched for outlier methylation marks when mutated by permutation analysis (see Methods), after Bonferroni-correction for the number of tested TFBSs (n = 133). For each factor, enrichment for differentially methylated CpGs between regulatory rare variants carriers and non-carriers is shown at the base of the bar in white. Significance threshold is indicated by the red-dashed vertical line.

We used permutation testing to assess the association between the disruption of TFBSs by rare SNVs and extreme CpG methylation (see Methods), and observed that 46 of the 133 tested TFBSs were significantly associated with extreme methylation values levels (Fig 2) (S3 and S4 Tables), suggesting that the sequence-specific binding of TFs to these 46 TFBSs could be involved in shaping local DNA methylation patterns. Amongst these TFBSs, 36 correspond to the canonical motif for well-known human TFs such as Yin and Yang 1 (YY1), CTCF and Specificity Protein 1 (SP1), while the remaining 10 correspond to anonymous motifs for which the potential binding factor has not yet been characterized.

As the direction of DNA methylation change can have different potential functional consequences on genome regulation, we next assessed the direction of the effect of rare SNVs on local DNA methylation changes. We observed a 1.45-fold enrichment for methylation gains across all tested SNV-TFBS pairs. For example, both CTCF and SP1 showed an enrichment for gains of methylation, which is in accordance with the known protective role of these two factors against DNA methylation [20].

### Minimal intersection between extreme DNA methylation values and DNA methylation quantitative trait loci

As variation in DNA methylation can also be driven by common genetic variation, we next assessed whether our results might be confounded by known meQTL or regions of haplotype-specific methylation (HSMs). We overlapped our results with CpGs where it is known that DNA methylation levels are associated with common genetic variation [21,22], and observed that only a very small fraction of CpG sites showing extreme DNA methylation and the disruption of TFBS by rare SNV (n = 189, or ~1% of the total) correspond to known meQTL or HSM regions. While these numbers do represent a significant enrichment for meQTL and HSM regions when compared to CpG sites that do not show outlier DNA methylation, these results indicate that the vast majority of our results are likely not driven by effects of common genetic variation.

### Disruption of functionally constrained sites within TF binding motifs is associated with larger methylation changes

Since the binding affinity for many TFs relies on the recognition of specific DNA sequences [23], a possible direct consequence of DNA sequence variation at TFBSs could be alterations of the TF-DNA interaction. The binding affinity preferences of TFs for a specific DNA sequence can be represented by position weight matrices (PWM). These matrices denote the nucleotide frequencies at each position of a given DNA sequence motif, allowing an estimation of the effect of motif variation on TF binding affinity in a site-specific manner.

**Fig 3:**
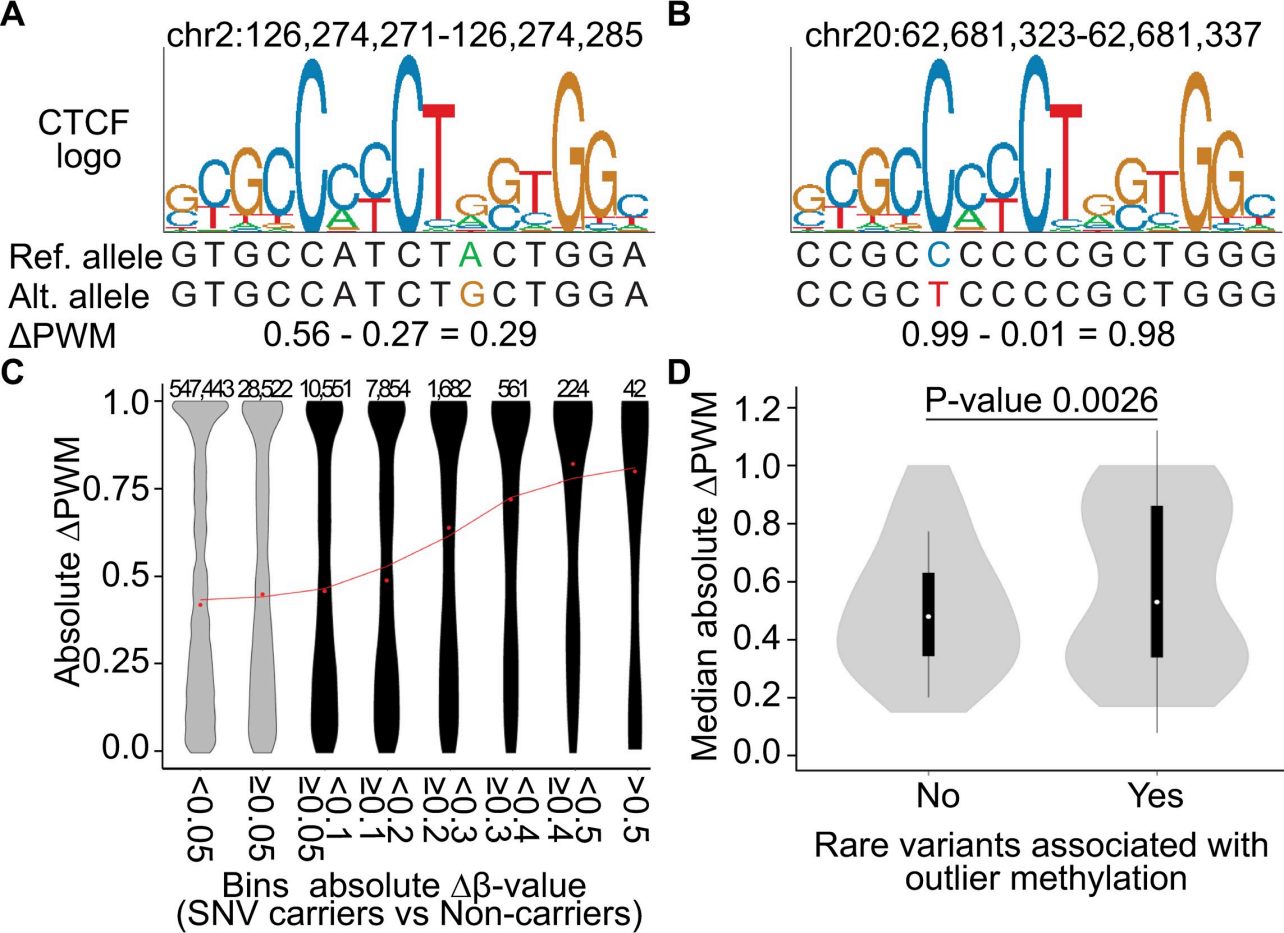
Mutation of highly constrained positions in TFBS motifs are associated with larger changes in DNA methylation. (A and B) Sequence logos showing SNVs within the consensus CTCF binding motif. While substitutions at degenerate positions (A) result in small changes to the position weight matrix score (ΔPWM), substitutions at highly conserved positions (B) cause large changes in ΔPWM. (C) Violin plots showing the distribution of ΔPWM scores for SNVs in TFBSs that are associated with different degrees of change in local DNA methylation. SNVs were stratified into eight different bins according to the degree of change in DNA methylation of associated CpGs (black and gray-filled plots corresponding to DNA methylation outlier and non-DNA methylation outlier, respectively). Red dots represent the median ΔPWM score for each bin, while the red line represents the smoothed median of three consecutive points. Above each violin is shown the number of associated CpGs per bin. (D) Violin plots showing the distribution of ΔPWM scores for SNVs that are associated versus those not associated with outlier methylation. The p-value is derived from the Wilcoxon matched-pairs signed rank test. Interquartile range (IQR) and median of the distribution are represented by boxes and white dots in the overlaid box plots, respectively. Whiskers represent upper/lower quartiles +/-1.5 IQR.

To infer the effect of rare SNV-TFBSs on TF-DNA binding, we computed separately for each SNV-TFBS the difference between the PWM score of the reference and alternate alleles (ΔPWM) [19] (http://hgdownload.soe.ucsc.edu/goldenPath/hg19/database/factorbookMotifPwm.txt.gz). Smaller values of ΔPWM correspond to SNVs at less constrained sites, which we hypothesized might be less likely to have an effect on TF binding efficiency (Fig 3A). Conversely, larger values of ΔPWM correspond to SNVs at more conserved positions within the motif that we hypothesized were more likely to alter the TF binding, and consequently impact DNA methylation (Fig 3B and 3C).

We analyzed 45 TFBSs, at which SNVs were associated with extreme DNA methylation changes and for which PWMs were available. Consistent with our hypothesis, we observed that rare SNVs disrupting those TFBSs associated with outlier methylation values showed higher ΔPWM scores compared to SNVs that are not associated with extreme methylation changes (Wilcoxon paired signed rank test, p = 0.0026) (Fig 3D). These results are consistent with rare variants altering local DNA methylation through altered binding of TFs to their motifs.

### Rare SNVs within TFBS exert stronger effects on local DNA methylation than SNVs within flanking regions

To confirm the key role of TFs in modulating local DNA methylation profiles, we compared the effect on local DNA methylation between SNV-TFBSs and SNVs that lie adjacent to but outside of annotated TFBS (SNV-noTFBS). Following the same approach as above, we assessed the effect on local DNA methylation for 571,137 different rare SNV-noTFBSs.

**Fig 4:**
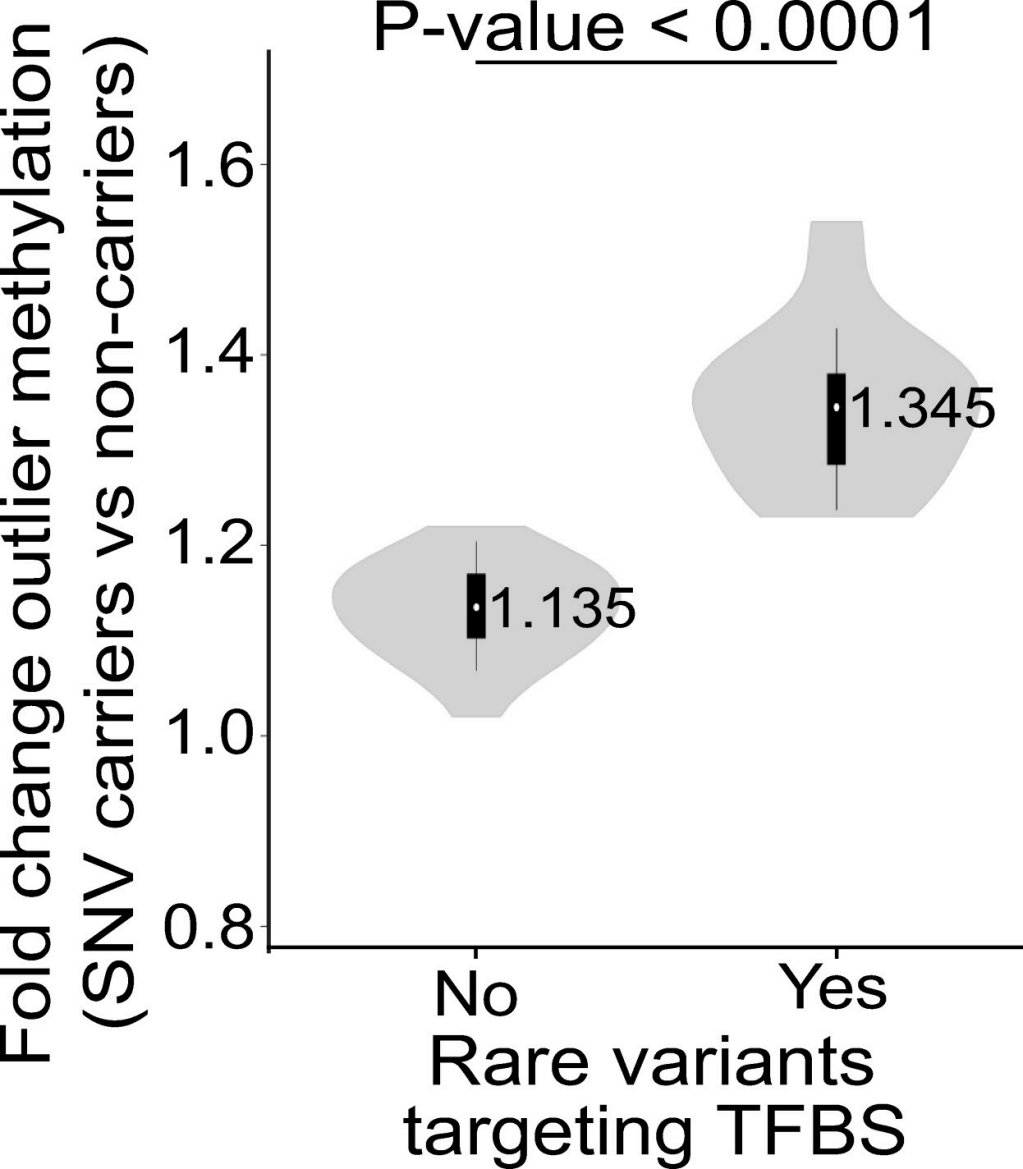
Mutations within TFBS are associated with larger effects on local DNA methylation. Violin plots showing enrichments of DNA methylation outliers associated with SNVs occurring within or in the flanks of TFBSs. Overlaid box plots as described in Fig 3. P-value derived from paired t-test.

Analogous to our previous analysis of rare SNV-TFBS, we observed a genome-wide enrichment for extreme methylation values for rare SNV-noTFBS, suggesting that local DNA methylation changes can also be driven by variants outside of annotated TFBSs. However, we observed that the effect of SNV-TFBSs on DNA methylation was 1.18 fold greater when compared to SNV-noTFBS (Paired t test, P-value<0.0001, Fig 4), indicating that rare genetic variants at TFBSs are enriched for effects on local epigenetics.

### Rare genetic variation within TFBS can influence DNA methylation over larger distances *in cis*

**Fig 5:**
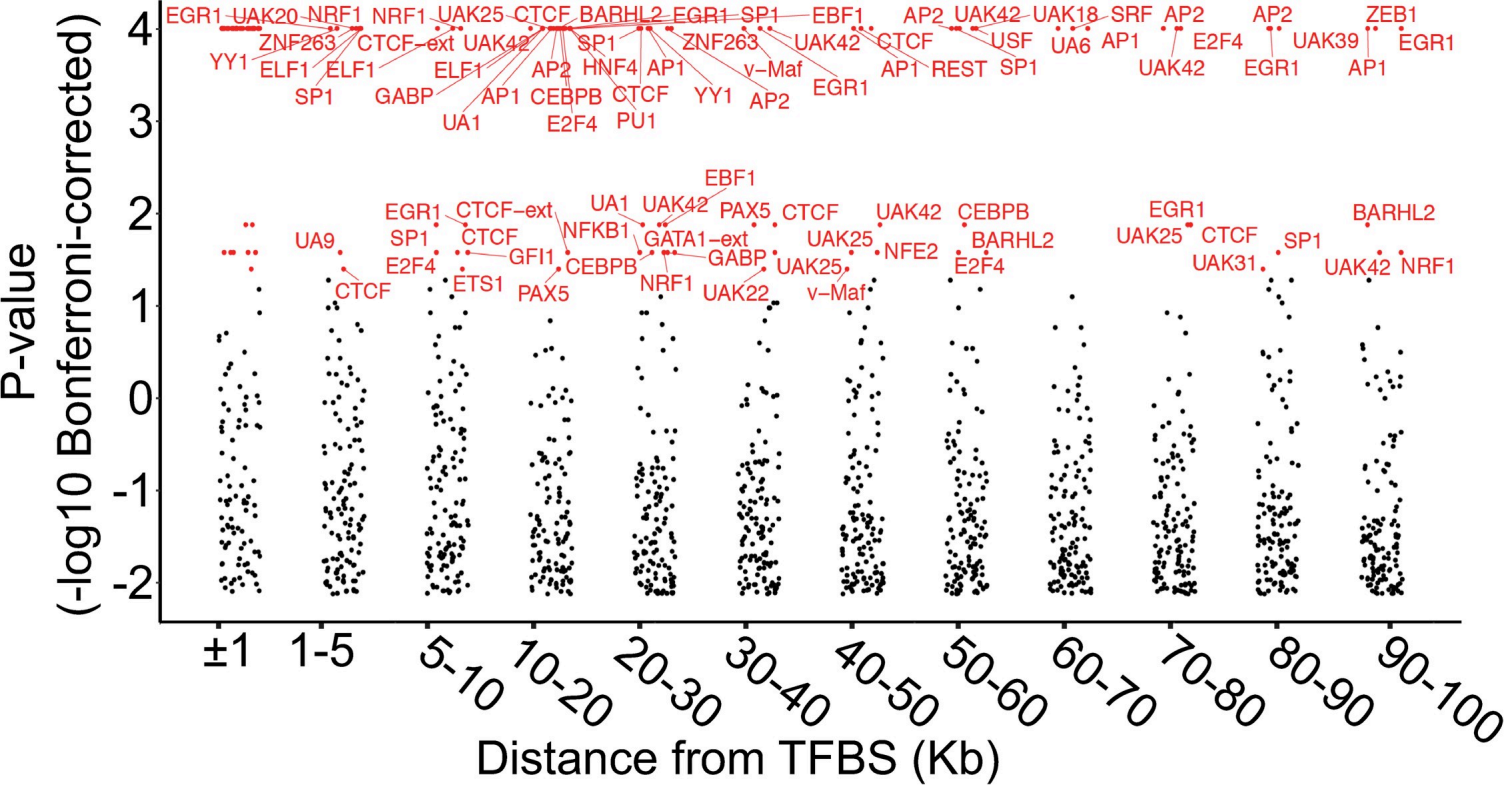
Long-range effects of regulatory rare variants on DNA methylation. For each TFBS containing a rare variant, CpGs located up to 100 kb away were grouped into bins according to their distance. Dots indicate p-values obtained for each TFBS by permutation testing after Bonferroni-correction for the number of TFBSs tested (n = 133). TFBSs with Bonferroni corrected p<0.05 in each bin are colored red, all others in black.

So far, we have shown that the disruption of TFBSs by rare SNVs is associated with altered DNA methylation profiles at the local level. However, we hypothesized that the effect of genetic variation on DNA methylation might also occur across wider regions [4,5]. To address this, we followed a similar approach to that described above, but selected β-values corresponding to CpGs located between 1–100 kb around each disrupted TFBS. We first binned β-values according to their separation from the disrupted TFBS, ranked and categorized them to define those showing extreme methylation, and then performed permutation testing for each TFBS per bin. After applying a multiple testing correction, we observed many significant associations between extreme DNA methylation and different TFBSs. However, there was a clear effect of physical distance, with the number of TFBS associations reducing markedly once separation of TFBS and CpG was >30 kb (Fig 5), suggesting that the majority of TF binding events modulate DNA methylation over relatively short distances (S5 Table).

### Disruption of DNA methylation by rare genetic variation at TFBS alters expression levels of nearby genes

Next, based on the key role of DNA methylation in the control of transcription [12], we explored the impact of DNA methylation changes associated with the disruption of TFBSs on the expression of nearby genes. As both DNA methylation and gene expression profiles are often tissue-specific, we used available cardiac DNA methylation and RNA sequencing (RNA-seq) expression profiles from 20 unrelated individuals for whom WGS data were also available.

**Fig 6:**
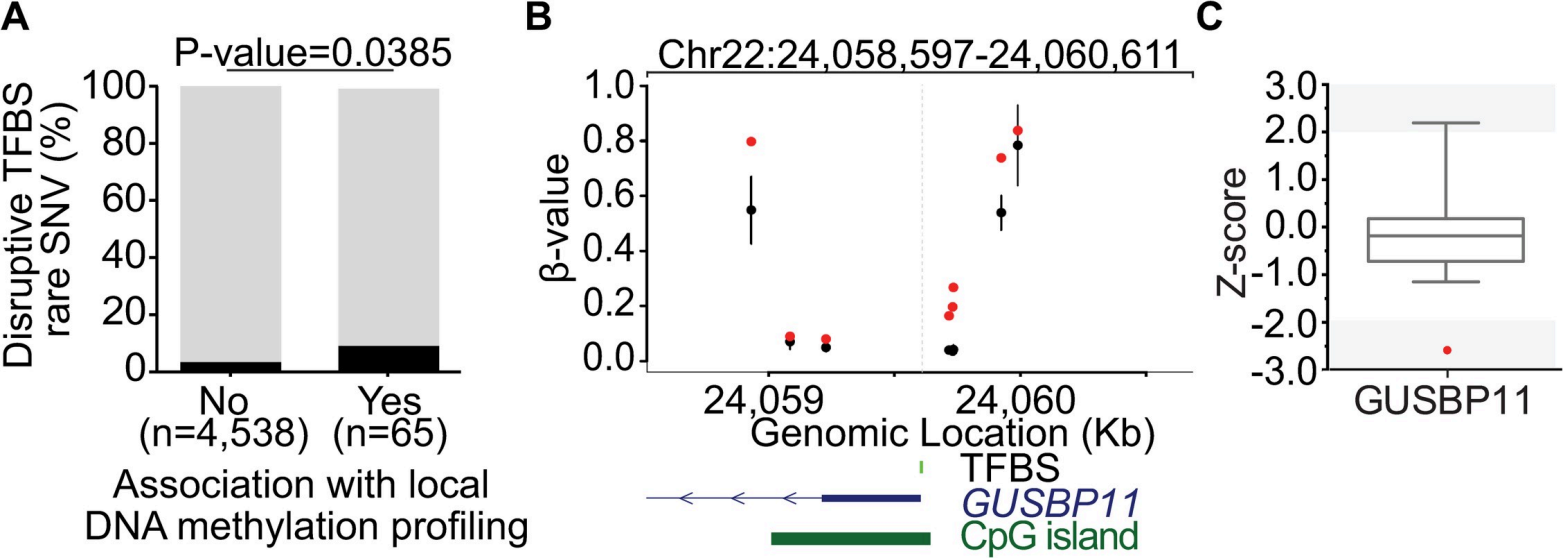
DNA methylation outliers linked with disruption of TFBS are enriched for gene expression outliers. **(A)** Bar plot showing the fraction of rare variants disrupting TFBSs that overlap promoters (TSS±2kb) of genes with outlier (black) and non-outlier expression (gray) in cardiac tissue. P-value derived from two-tailed Fisher’s exact test. **(B)** Plot showing DNA methylation profile at chr22:24,058,597–24,060,611 (UA4 binding site ±1 kb) genomic region. DNA methylation values are shown for an individual with a rare variant at chr22:24,059,610 (red dots), while black dots and bars represents the mean ±2 standard deviation of controls. Position of rare variant that disrupts the UA4 TFBS motif is depicted by vertical gray dashed line. Expression of the nearby *GUSBP11* gene **(C)** is down-regulated in this individual carrying the rare variant (red dot) compared to controls. Expression of *GUSBP11* in controls is represented by box plot, with gray shaded areas indicating outlier expression levels.

After quality control (QC) procedures and filtering (see Methods), we identified 12,903 rare SNVs that overlapped with 17,620 TFBS motifs. From these, we selected 4,944 rare SNVs that disrupted TFBSs within gene promoter regions (TSS±2kb), intersected these with normalized gene expression data, allowing evaluation of the impact of 3,978 rare regulatory SNVs on 3,478 independent transcripts. In comparison to gene promoters with a normal DNA methylation profile, this analysis revealed a 2.58-fold enrichment for extreme expression levels (>2 Z-scores from the mean) associated with genes with altered promoter methylation and a rare SNV in a nearby TFBS (p = 0.0363, two-tailed Fisher’s exact test, Fig 6A) (S6 Table). An example is shown in Fig 6B and 6C, where an individual with a rare variant within the canonical TFBS for UA4 (predicted to be THAP1) shows increased methylation levels at the promoter of the nearby *GUSBP11* gene (Fig 6B). Consistent with the known repressive effects of DNA methylation at gene promoters, this is associated with reduced expression of *GUSBP11* (Fig 6C). This result suggests that a fraction of rare genetic variation at TFBS contribute to local transcriptional regulation by modulating DNA methylation profiles.

## Discussion

Here, we evaluated the association of rare regulatory genetic variation on DNA methylation profiles by focusing on extreme methylation values in a cohort of 247 individuals for whom both WGS and methylation profiles were available. Our results indicate that a fraction of rare SNV-TFBSs are associated with outlier DNA methylation profiles, which we suggest is potentially due to alterations in TF binding, and these events are associated with altered expression of nearby genes. Furthermore, our results highlight the value of incorporating DNA methylation data to interpret the functional consequences of rare regulatory genetic variation.

Analogous to our results, it has been recently shown that rare genetic variation is sometimes associated with extreme expression levels of nearby genes [9]. Interestingly, these regions are strongly enriched for variants at CpG-rich promoter regions and TFBSs. Based on our results, we hypothesize that a certain fraction of this altered gene expression might be due to changes in DNA methylation profiles as a consequence of rare genetic variation, especially involving SNVs within TFBSs.

We observed that a total of 46 canonical TFBSs are significantly associated with local DNA methylation patterns. Consistent with previous reports showing that genetic variation at CTCF binding sites have been involved in modulating, creating and propagating DNA methylation profiles in mammals [16,17,21], we observed a significant association between local DNA methylation profiles and the presence of rare SNVs in CTCF-binding sites. In addition to CTCF, disruption of the binding sites of ten other TFs in our list of 46 has recently been shown to cause alterations of local allelic DNA methylation [24]. In line with our results, Onuchic et al. showed that the resulting DNA methylation patterns are more often linked to rare, rather than common, genetic variation, reinforcing our approach of focusing on rare genetic variation. Altogether, these studies not only validate our approach to detect rare SNV-TFBS-directed DNA methylation but also suggest that the modulation of local DNA methylation by TFs represents a general mechanism involved in the regulation of genome function, rather than being limited to a few TFs.

In addition to local effects on DNA methylation, we also observed that, in some instances, rare genetic variation at TFBSs is also associated with changes in DNA methylation up to distances of 100 kb. This is in agreement with the potential role of some TFs in DNA loop formation [25–27], which can bring regions located hundreds of kilobases away into contact. For instance, we observed that CTCF, a structural protein involved in the establishment and maintenance of the 3D conformation of the genome [27], is consistently associated with extreme methylation values up to 50 kb from the disrupted TFBS. Similarly to CTCF, we also observed that AP-1 has effects on DNA methylation over large distances; notably, AP-1 has also been implicated in the formation of DNA loops during development [28].

According to our results, TFs seem to play a key role in preferentially dictating local DNA methylation profiles. Nevertheless, we still observed an enrichment for extreme methylation values associated with rare genetic variation outside of TFBSs. An explanation for this enrichment could be that, despite being outside of these regulatory elements, SNVs can still have an impact on TF activity, as recently shown by both computational and experimental approaches [29–32]. For instance, mutations at positions immediately adjacent to GATA1 binding motifs result in reduced binding of this TF [31], indicating that TF binding is not only regulated by genetic variation within the TF motif. Another possible explanation could be that these variants might lie within unannotated binding motifs for TFs that were not included in this study. For example, it is estimated that approximately one third of human TFs lack DNA-binding affinity models [33]. Furthermore, we cannot rule out that local DNA methylation profiles could be modulated by additional mechanisms.

The mechanisms by which TF binding mediates local DNA methylation are still unclear. Since TFs lack enzymatic activity, it is unlikely that they directly catalyze the gain or loss of DNA methylation at CpG sites. One possible mechanism would involve the formation of complexes with DNA methylation machinery, such as DNA methyltransferases (DNMTs) and ten-eleven translocation (TET) proteins [34–36]. For example, it has recently been shown that nuclear receptor subfamily 6 group A member 1 (Nr6a1) can interact with Dnmt3a and Dnmt3b proteins and induce DNA methylation at the *Oct4* gene promoter that contains Nr6a1 binding sites [36,37]. Conversely, local DNA demethylation could result from the interaction between TFs and TET proteins in mammalian cells [34,35]. However, mutations at CTCF binding sites result in abnormal methylation of the maternal unmethylated allele of the H19 Imprinted Control Region in mice [38], suggesting that the binding of the TF on the DNA itself can protect against DNA methylation, likely by preventing accessibility to DNMTs at these sites. Altogether these data provide a possible mechanistic link between the alteration of TFBS by rare SNVs and the resulting DNA methylation profile.

While previous studies have shown that epigenetic changes due to regulatory mutations often lead to regional changes affecting clusters of multiple CpGs, it should be noted that for the majority of SNV-TFBS in our study (74%), we identified only a single associated outlier CpG. There are several possible explanations for this observation: (i) the EPIC array that we used to interrogate DNA methylation only has relatively sparse genomic coverage, sampling only a small subset of CpGs at most genomic loci; (ii) due to the large number of SNV-TFBS we interrogated, some of the pairwise associations with outlier methylation occurred by chance, and (iii) differential methylation of single CpGs might sometimes result from inaccurate measurements due to poorly performing probes or other technical artefacts, and does not represent true epigenetic variation [39]. To minimize this latter possibility, we performed pre-processing of the methylation dataset including the removal of potentially confounded probes and normalization. Despite this, we observed a 1.7-fold enrichment (p<0.0001, two-tailed Fisher’s Exact test) for multi-mapping probes reported by Pidsley et al. [40] in our set of CpGs showing extreme DNA methylation. However, it is important to note that these CpGs only account for a very small fraction of the overall set of outlier CpGs we reported (approximately 500, or <3% of outlier CpGs). Therefore, although we cannot rule out that a proportion of our results might be driven by technical artefacts, we believe that this fraction is likely very small and does not affect the overall conclusions of this study.

## Conclusions

Our data suggest that a meaningful fraction of rare genetic variation at TFBSs can play a role in shaping DNA methylation profiles in cis, which in turn can result in altered expression of the nearby genes. Furthermore, we provide a rationale for integrating DNA methylation data to identify genuine functional genetic variation from the broader genetic background, which represents a current challenge in human genetics.

## Materials and methods

### Sample description

A total of 249 unrelated individuals with paired data for WGS and EPIC DNA methylation profiling of peripheral blood collected at the time of enrollment were selected from the cohort collected via Pediatric Cardiac Genomic Consortium (PCGC) [41]. An extensive description of PCGC cases as well as further details about sample collection can be found in a summary publications released by the PCGC [41,42]. Briefly, our cohort comprises 249 individuals, aged from newborn to 47 years (mean 8.2 years) and diagnosed with a range of congenital heart defects, with conotruncal and left-sided obstructive lesions being the two most common diagnoses (S7 Table).

### Ethics statement

This research was approved by the Institutional Review Board of the Icahn School of Medicine at Mount Sinai under IRB #17–01980. All samples were collected after obtaining written informed consent from each participant or their parents for broad genomic studies approved by the Institutional Review Boards of Boston’s Children’s Hospital, Brigham and Women’s Hospital, Children’s Hospital of Los Angeles, Children’s Hospital of Philadelphia, Columbia University Medical Center, Great Ormond Street Hospital, Icahn School of Medicine at Mount Sinai, Rochester School of Medicine and Dentistry, Steven and Alexandra Cohen Children’s Medical Center of New York and Yale School of Medicine. Further details of ethical issues regarding PCGC samples, including patient privacy, data storage, and return of results to participants, can be found in a summary publication released by the PCGC [41].

### Illumina sequencing, quality control and variant filtering

After TruSeq DNA PCR-free (Illumina Inc., San Diego, CA, USA) library preparation, WGS was performed on genomic DNA isolated from peripheral blood at Baylor College of Medicine (Houston, TX, USA) using Illumina HiSeq instrument to obtain an average of 36-fold genome coverage (range from 25- to 39-fold). Paired-end reads were aligned to human reference genome (GRCh37/hg19) using BWA-mem [43]. SNVs were called from aligned reads using GATK (v2.7) as previously described [44–46].

After applying filters for variant quality (GATK VQSR, ≥10 total reads, genotype quality score ≥30 and alternate allele fraction between 0.2–0.8 and ≥0.9 for heterozygous and homozygous SNPs, respectively), we retained for further analysis those SNVs with MAF ≤1% in the 1000 Genomes Project (1000G) [6] and Genome Aggregation Database (GnomAD) [7] (http://gnomad.broadinstitute.org/about) databases, as well as in our cohort (n = 247).

### Illumina Infinium MethylationEPIC BeadChip array profiling

Genome-wide DNA methylation profiling was performed on genomic DNA isolated from peripheral blood using the EPIC array (Illumina, Inc., San Diego, CA, USA) according to Infinium HD Methylation Assay Protocol (IIlumina). Methylation levels are given as β-values, representing the ratio of intensities between the methylated and unmethylated signals. Resulting β-values range from 0 for completely unmethylated to 1 for completely methylated.

For every sample, raw data files with β-values, intensity values per channel, and detection p-values per probe were obtained from the New York Genome Center. Before processing, QC of raw data was performed comprising a gender check comparing X and Y-chromosome data against reported sample gender, principal component analysis (PCA) plots and density plots of *M* values. Based on these steps, two samples were excluded from downstream analysis, as they were clear outliers on PCA plots. To prevent technical biases that could influence methylation measurements, we initially excluded EPIC probes with (i) internal common SNPs (MAF ≥5% in 1000G database) within the last 5 bases of the 3΄ end of the probe or (ii) non-unique mapping to the bisulfite-converted genome. Due to gender differences on the sex chromosomes, only β-values of autosomal probes were utilized in downstream processing. Raw β-values for 822,016 EPIC probes for the remaining samples (n = 247) were background and color corrected and quantile normalized using *lumi* and *methylumi* R packages [47]. Finally, Infinium probe design biases were corrected using BMIQ [48].

Since DNA methylation patterns are often tissue-specific and blood consists of multiple different cell-types [12], resulting β-values can simply reflect differences in cell-type composition among samples. To prevent this potential confounder, we first estimated the fraction of six blood cell types (CD4+, CD8+, B and natural killer cells, granulocytes and monocytes) in our samples applying Houseman algorithm [49] and correlated these estimates with β-values of each probe. We observed that the probes most significantly correlated with cell-type fraction (Bonferroni corrected P-value for number of probes <0.05) showed larger methylation differences, suggesting that heterogeneity in white blood cells across our samples had the potential to confound our DNA methylation measurements (S1 Fig, right panel). Subsequently, this bias was removed by excluding most highly correlated probes with every cell fraction up to 5% of the total number of processed probes on the EPIC array (S1 Fig, left panel). Following this approach, a total of 76,776 potentially cell-type confounded EPIC probes were excluded from our analysis. Furthermore, we also excluded EPIC probes with rare SNV-TFBSs present within the probe, at the interrogated CpG or its adjacent base.

### Enrichment of extreme DNA methylation values due to the disruption of TFBS by rare SNVs

For this analysis, only autosomal SNVs derived from WGS data from 247 unrelated with minor allele frequency (MAF) ≤1% in GnomAD and 1000G databases as well as in our cohort (n = 247) (Number of rare SNVs per individual, mean = 84,245, max = 320,786, min = 47,391) were included. Furthermore, to capture rare events, SNVs that were present in >2 individuals were also excluded. Based on previous observations about the potential role of TFs in shaping DNA methylation profiles and how this process can be affected by genetic variation at their binding sites [15–17], we focused on rare SNVs overlapping TFBSs. We used a catalogue of 2,298,872 motifs for 133 human TFBSs identified from ChIP-Seq peaks generated by the ENCODE project [18,19,50]. Finally, to avoid redundancy in our analysis, we merged overlapping TFBSs for the same TF that share the same SNV, respectively. This resulted in a total of 131,357 SNVs that overlap 188,226 TFBSs.

After performing standard QC and processing (See above), we extracted β-values corresponding to EPIC probes located within the proximity of the SNV-disrupted TFBS, *i*.*e*., the SNV-disrupted TFBSs and their ±1-kb-extended flanks, and ranked these from lowest (1) to highest (247). As a result of intersecting our genotype and DNA methylation datasets, we identified a total of 91,356 rare SNV across 120,096 different binding motifs that were informative for DNA methylation, *i*.*e*., EPIC probes mapping within 1-kb-extended flanks of SNV-targeted TFBS (217,578 different EPIC probes). Bedtools (v2.27) was used to intersect rare SNVs derived from WGS with 2,298,872 motifs instances corresponding to 133 canonical TFBSs, obtained from the UCSC Genome Browser track “Transcription Factor ChIP-seq (161 factors) from ENCODE with Factorbook Motifs”. These motifs (size 8–24 bp) have been predicted using data obtained from ChIP-Seq experiments on 119 human TFs in multiple cell lines generated by the ENCODE Consortium [19]. (http://hgdownload.soe.ucsc.edu/goldenPath/hg19/database/factorbookMotifPos.txt.gz, March 16^th^ 2014 release) [18,19].

To avoid redundancy in our analysis, overlapping motif instances of the same TFBS with a single SNV were merged into a single motif using bedtools groupby tool (v2.27). CpGs of interest, *i*.*e*. those within 1 kb flanking each TFBS, were identified using bedtools (v2.27). Next, the selected CpGs were ranked by increasing β-value and annotated for our extreme CpG methylation criteria, defined as within the 5% tails of rank distribution and a minimum absolute difference of 0.05 in β-value between SNV-TFBS carriers and non-carriers. Individuals were also annotated according to the presence (SNV carrier) or absence (SNV non-carrier) of a given SNV-TFBS. Enrichment for extreme methylation values was calculated as the ratio of the fraction of extreme CpG methylation values in the group of SNV-carriers over the fraction of extreme methylation values in the group of SNV-non-carriers. In order to assess statistically the ability of SNV-TFBSs to modulate local DNA methylation profiles, we performed 10,000 permutations where we randomized our data and re-calculated the ratio of extreme methylation values between the groups of SNV carriers and non-carriers. P-values were calculated as the fraction of permutations where this ratio was equal to or exceeded the observed data. We then applied a Bonferroni correction for the number of different TFBS tested (n = 133).

A similar approach was carried out to determine the effect of the disruption of TFBSs by rare SNVs on DNA methylation over distances >1 kb. However, here we first binned CpGs according to separation from the TFBS, and permutation testing was performed separately for each TFBS and bin, as described above. For each bin, resulting p-values from the permutation test were Bonferroni corrected for the number of different tested TFBSs (n = 133).

### Identification of differentially methylated regions

We screened for DMRs in each sample using a sliding window algorithm, similar to that described in [16]. Briefly, for each individual sample, this algorithm searches for genomic intervals where, within a 1 Kb window:

- at least 3 probes each have β values above the 95th percentile of the cohort distribution for that probe, and are ≥0.1 above the cohort mean (Hypermethylation).
- at least 3 probes each have β values below the 5th percentile of the cohort distribution for that probe, and are ≤0.1 below the cohort mean (Hypomethylation).

Because our algorithm is only able to detect DMRs at genomic intervals where ≥3 probes within 1 kb are assayed by the EPIC array, we intersected our DMR list with the set of SNV-TFBS that had at least one sample with an overlapping mutation associated with outlier methylation, and which had 3 or more CpGs located within 1kb (n = 16,708).

### Enrichment analysis for meQTL and HSMs

As the meQTL reported by Do *et al*. [21] are based on CpG sites present on the Infinium Human Methylation 450K BeadChip (450K), we first selected 145,041 CpG probes that are present within the proximity of mutated TFBS (±1Kb either side) and are present on both the 450K and EPIC array platforms. After categorizing these according to their presence in the list of meQTL provided by Do *et al*. [21] and their methylation profile (extreme and non-extreme), we estimated enrichment of these probes for meQTL according to their methylation profile.

Similarly, to assess the potential impact of HSMs on our results, we first categorized the set of 217,578 EPIC probes included in our study according to their presence within HSMs and their methylation profile (extreme and non-extreme). Subsequently, we estimated the enrichment of them for these genomic intervals according to their methylation profile. Statistical significance was tested by two-tailed Fisher’s Exact test.

### Positional impact of genetic variants within TF motifs

The potential effect of each base substitution on predicted DNA-binding was estimated by computing the absolute difference in Position Weight Matrix (ΔPWM) scores of the two alleles at the variant site using available PWMs (http://hgdownload.soe.ucsc.edu/goldenPath/hg19/database/factorbookMotifPwm.txt.gz) (March 16^th^ 2014 release). This file includes PWMs for 131 different canonical TFBSs generated from ChIP-Seq data generated by the ENCODE project [19].

We analyzed 45 TFBSs, at which SNVs were associated with extreme DNA methylation changes and for which PWMs were available. Prior to computing ΔPWM scores, for each tested TFBS we categorized rare SNV-TFBSs into two groups according to their association to extreme methylation values. One group included those SNV-TFBSs associated with local extreme methylation values and another consisting of the remaining SNV-TFBSs. In cases where multiple EPIC probes overlap the same 1-kb-extended TFBS interval, we considered rare SNV-TFBSs as associated with local extreme methylation when at least 1/3 of the CpGs fulfilled our criteria for extreme CpG methylation. Furthermore, to avoid ambiguity in our PWM-based approach, we only selected those variants within isolated TFBSs, *i*.*e*., that has not been previously merged with any overlapping TFBS. After this selection, a total of 62,400 (66.1%) rare SNV-TFBSs were included in this analysis. We next calculated the ΔPWM score between the alleles for the selected SNV and compared the median of the ΔPWM score between the two groups. For each TFBS, the median of the absolute ΔPWM scores was calculated for each SNV-TFBS group and statistically compared using a Wilcoxon matched-pairs signed rank test.

### Functional constraint and effect size on DNA methylation

Each CpG was annotated with the absolute ΔPWM score based on the *cis*-linked SNV-TFBS. CpGs were binned according to their association with extreme CpG methylation values, and the magnitude of the methylation change between SNV-carriers and non-carriers. Pearson correlation between the median values of absolute ΔPWM scores and magnitude of methylation change was computed across each bin.

### Rare TFBS vs non-TFBS rare SNVs effect on DNA methylation at nearby CpG sites

Rare SNVs that were present within 1-kb flanks of TFBS but did not overlap an annotated TFBS were identified by bedtools (v2.27). After ranking CpGs based on their β-values and annotating for extreme CpG methylation, we calculated enrichment for extreme methylation in individuals carrying SNVs outside TFBSs. A paired t-test was used for matched chromosomal enrichment for extreme CpG methylation values between SNV–TFBS and SNV outside.

### Functional consequences of rare genetic variation at TFBS in heart tissues

#### Selection of rare SNVs

Similar to our previous analysis in blood, only rare SNVs derived from WGS with MAF ≤1% in the 1000G [6] and GnomAD [7] databases as well as in the PCGC cohort were retained for further analysis. These SNVs were intersected with motifs of 133 canonical TFBSs using bedtools (v2.27). Furthermore, overlapping motif instances for the same TFBS that are targeted for the same SNV were merged using bedtools groupby tool (v2.27).

#### RNA-Sequencing

Twenty four transcriptome profiles derived from RNA-Seq of ventricle tissue were collected from Richter F. *et al*.[51]. Briefly, RNA extracted from cardiac tissues that were either snap-frozen or conserved in *RNA-later* from 327 participants who underwent cardiac surgery were sequenced on the Illumina Hi-Seq X Ten or NextSeq instrument using 50-bp paired-end sequencing. After sequence reads were aligned to the hg19 reference genome using Subread [52], read counts were calculated per gene using featureCounts [53]. Genes showing a mean of ≥1 read per kb per million mapped reads (RPKM) across the samples were considered as expressed in cardiac tissue. Genes below this threshold were excluded from further analysis. For the remaining genes, expression levels were corrected for known covariates (tissue, library preparation, sequencing platform, tissue storage, age, and gender) and subsequently, converted to Z-scores. Expression levels >2 Z-scores from the mean were considered as outliers.

Although PCA plots for gene expression profiles did not show high variance across our samples, four samples were excluded from this analysis as they display a significantly higher number of genes showing outlier expression as compared to the remaining samples (S2 Fig).

#### Cardiac methylation profiling

Genome-wide DNA methylation profiling was performed on genomic DNA isolated from ventricular tissues obtained from 25 individuals who underwent cardiac surgery. One sample was excluded from this analysis as there was a mismatch between array-inferred gender utilizing data from the sex chromosomes and the reported gender. Raw data files with β-values, intensity values per channel, and detection p-values per probe were processed as previously described for blood DNA methylation profiling, without controlling for leukocyte distribution.

A total of 20 individuals with available WGS data, cardiac RNA sequencing (RNA-Seq), and EPIC DNA methylation profiles were selected. Processed β-values for CpGs present within the targeted TFBS by SNV or their 1-kb flanks were selected for downstream analysis. Unlike our previous analysis in blood samples, due to the smaller size of our cohort we considered extreme CpG methylation values as those at the top (1) or bottom (20) of the rank distribution and showing an absolute minimum β-value difference of 0.05 between SNV carriers and non-carriers. Subsequently, SNV-TFBSs were categorized according to the extreme CpG methylation content within its flank. In cases where there were multiple CpGs within 1 kb of a TFBS, we considered this SNV-TFBS associated with extreme methylation values when at least one third of the CpGs fulfilled our criteria for extreme methylation.

After DNA methylation and gene expression outlier annotation, SNV-TFBSs were annotated with the closest RefSeq gene using bedtools (v2.27). Only those SNV-TFBSs that fell within gene promoter (TSS±2kb) of the closest gene were selected. Expression and DNA methylation data were intersected using the dplyr R package and, subsequently, an enrichment analysis for expression outliers between SNV-TFBSs associated with extreme methylation and SNV-TFBSs that were not associated with extreme methylation was performed. Statistical significance was tested by two-tailed Fisher’s exact test.

## Supporting information

S1 Fig Blood cell-type specific composition influences DNA methylation. Correlation between DNA methylation values and blood cell-type specific fraction was calculated for each CpG site present on the EPIC Illumina using Houseman algorithm [49]. CpG sites mapping within TFBSs that overlap with rare SNVs or their flanks (±1 kb) were ranked according to their correlation level with cell type fraction. These CpG sites were plotted in decreasing rank against the absolute β-value difference between individual carrying tested SNV and controls. Individual CpG sites are represented by dots and they are displayed in decreasing order according to their correlation with specific blood cell–type. Black and red-filled dots represent ranks outside and inside of 5% tails of the DNA methylation ranks distribution, respectively. The white curve represents smoothed line.
(EPS)

S2 Fig Principal component analysis (PCA). **(A)** based on 13,190 genes expressed in ventricular tissue obtained from 24 individuals and **(B)** bar plot showing the number of genes showing outlier expression (expression value above/below ±2 Z-score) in these individuals.
(EPS)

S1 Table TFBS included in association study. (XLSX)

S2 Table List of differentially methylated regions due to the disruption of TFBS by rare SNVs. (XLSX)

S3 Table Permutation test for individual TFBSs to test local effect of the disruption of TFBSs by rare SNVs on DNA methylation. (XLSX)

S4 Table List of EPIC probes associated with extreme DNA methylation upon disruption of TFBSs by rare SNVs. (XLSX)

S5 Table Wider effect on DNA methylation profile of rare SNVs that disrupt TFBSs. (XLSX)

S6 Table Genes showing outlier expression associated to local extreme DNA methylation profile due to the disruption of TFBSs by rare SNVs in ventricular tissues. (XLSX)

S7 Table Phenotype of individuals included in the study. (XLSX)
